# Direct comparison of multilayer left ventricular global longitudinal strain using CMR feature tracking and speckle tracking echocardiography

**DOI:** 10.1186/s12872-021-01916-8

**Published:** 2021-02-19

**Authors:** Saikrishna Ananthapadmanabhan, Giau Vo, Tuan Nguyen, Hany Dimitri, James Otton

**Affiliations:** 1 Faculty of Medicine, University of New South Wales, Sydney, 2052 Australia; 2 Cardiology Department, Liverpool Hospital, Liverpool, Sydney, 2170 Australia

**Keywords:** Cardiac magnetic resonance, Feature tracking, Speckle tracking echocardiography, Multilayer

## Abstract

**Background:**

Cardiac magnetic resonance feature tracking (CMR-FT) and speckle tracking echocardiography (STE) are well-established strain imaging modalities. Multilayer strain measurement permits independent assessment of endocardial and epicardial strain. This novel and layer specific approach to evaluating myocardial deformation parameters may provide greater insight into cardiac contractility when compared to whole-layer strain analysis. The aim of this study is to validate CMR-FT as a tool for multilayer strain analysis by providing a direct comparison between multilayer global longitudinal strain (GLS) values between CMR-FT and STE.

**Methods:**

We studied 100 patients who had an acute myocardial infarction (AMI), who underwent CMR imaging and echocardiogram at baseline and follow-up (48 ± 13 days). Dedicated tissue tracking software was used to analyse single- and multi-layer GLS values for CMR-FT and STE.

**Results:**

Correlation coefficients for CMR-FT and STE were 0.685, 0.687, and 0.660 for endocardial, epicardial, and whole-layer GLS respectively (all p < 0.001). Bland Altman analysis showed good inter-modality agreement with minimal bias. The absolute limits of agreement in our study were 6.4, 5.9, and 5.5 for endocardial, whole-layer, and epicardial GLS respectively. Absolute biases were 1.79, 0.80, and 0.98 respectively. Intraclass correlation coefficient (ICC) values showed moderate agreement with values of 0.626, 0.632, and 0.671 respectively (all p < 0.001).

**Conclusion:**

There is good inter-modality agreement between CMR-FT and STE for whole-layer, endocardial, and epicardial GLS, and although values should not be used interchangeably our study demonstrates that CMR-FT is a viable imaging modality for multilayer strain

## Introduction

Strain is a quantitative measure of myocardial deformation, varied with respect to the directional aspect of motion, either radial, circumferential, or longitudinal. It can be described as global, involving the whole myocardium, or segmental, most commonly as defined by the standard 18 segmental model of the heart [1]. Global longitudinal strain is the most studied parameter in the literature and has been shown to be an important parameter in investigating cardiac function [2] that can provide additive diagnostic and prognostic value in various cardiac pathologies. A systematic review of 16 articles (n = 5721) by Kalam et al. showed that GLS has superior prognostic value compared to LVEF in predicting major adverse cardiac events [3]. This is due to greater sensitivity to subtle changes of functional impairment in early disease states when compared to conventional echocardiographic parameters.

Cardiovascular magnetic resonance feature tracking (CMR-FT) has been shown to be a reliable and robust technique for the quantification of myocardial strain [4]. It is a form of tissue tracking technology similar to speckle tracking echocardiography, and permits offline analysis of standard CMR cine steady-state free precession images. It involves tracking of the endocardial and epicardial borders and pattern tracking of anatomic features and voxel motion within the myocardial tissue. Whole layer CMR-FT derived GLS has been validated against CMR myocardial tagging, regarded as the gold standard of strain analysis [5]. CMR-FT has advantages over CMR myocardial tagging, most notably faster analysis times and more feasible acquisition and post-processing procedures [4]. It is gaining popularity in clinical practice in the evaluation of cardiac pathologies including coronary artery disease and cardiomyopathies. Furthermore, comparative studies between STE and CMR-FT for whole-layer GLS have shown excellent correlation and good intermodality agreement [6, 7]. Studies have advocated its use, especially in patients with suboptimal echocardiogram image quality, due to its feasibility and accuracy [6].

Novel developments in strain imaging techniques have led to the advancement of multilayer or layer-specific analysis. This facilitates independent evaluation of myocardial contractility at the endocardial, mid-myocardial, and epicardial levels. The implications of this technique in clinical practice is promising. Many cardiac pathologies, both valvular [8] and myocardial [9, 10] are known to have a heterogeneous and non-uniform impact on the myocardium. For example, subendocardial tissue is affected earlier in hypertrophic obstructive cardiomyopathy (HOCM) [11] and is affected to a greater degree in comparison to subepicardial tissue in non-ST elevation myocardial infarction [10].

Assessment of global or segmental strain with a layer-specific approach gives rise to the possibility of localising disease and detecting pathology earlier within the disease course. There are potential implications in utilising strain for risk stratification of patients with cardiac pathology. However, more research is required in this field before implementation in routine clinical and academic applications. Validation studies of whole-layer strain measurements for both STE and CMR-FT are available in the current literature, however those for multilayer strain are lacking. Ishizu et al. provided experimental validation of multilayer STE-derived measurements of global circumferential strain (GCS) against sonomicrometry in a study of 11 sheep showing acceptable intraclass correlations for endocardial, mid-myocardial, and epicardial GCS [12]. Liu et al. performed the first multilayer strain study using a cine-MRI based algorithm to demonstrate the transmural distribution patterns of GLS and GCS in healthy volunteers [13]. This study aims to investigate the inter-modality agreement for whole- and multi-layer GLS with respect to CMR-FT and STE.

## Methodology

### Subject recruitment

Ethical approval for this study was obtained from the Concord Human Research Ethics Committee.

The study involved a cohort of 100 patients who presented to Liverpool Hospital with an AMI. The recruitment criteria included standard ECG criteria for acute ST elevation myocardial infarction (STEMI) with presence of ST elevation in 2 contiguous leads ≥ 2 mm for leads V1-V3, or ≥ 1 mm for other leads, or for posterior AMI ≥ 1 mm ST depression for leads V2-V3. The patients were treated by primary PCI, thrombolysis, or rescue PCI. All patients had a verified total occlusion or severe stenosis after reperfusion of a major epicardial vessel on coronary angiography. Standard clinical exclusion criteria included patients with end stage renal failure, allergy to Gadolinium contrast, prior valvular cardiothoracic surgery or congenital heart disease, MRI exclusion criteria, and age ≤ 18 years and ≥ 85 years. The study cohort underwent both CMR imaging and echocardiogram at baseline and follow-up, with both imaging modalities occurring on the same day. Follow-up imaging occurred at 35–49 days post initial admission for STEMI. We performed strain analysis on both the baseline and follow-up data and combined the data set. As such, the endocardial data involves endocardial strain in the baseline and follow-up CMR scans (n = 100 patients, 200 scans) compared to the baseline and follow-up STE scans (n = 100 patients, 200 scans). The same is applicable for the whole-layer and epicardial GLS scans.

### CMR Acquisition and CMR-FT analysis

All CMR measurements were performed in a standard supine position using a commercially available MRI at 1.5 T (Siemens Symphony). Images were acquired at 8 mm slice thickness with a typical in-plane resolution of 1.5625 mm × 1.5625 mm and 25 phases per cardiac cycle. A multi-technique imaging protocol was implemented with an ECG-gated steady state free-precession cine sequence taken during periods of breath holding taken in the following planes: left ventricular (LV) 2-chmaber, 3-chamber, and 4-chamber in the long axis plane and equidistant short-axis planes completely covering both ventricles and including basal, mid-ventricular, and apical segments. Each subject had a complete data set with short-axis stack and three long axis view. We used this same methodology for CMR-FT acquisition in a previous paper [14].

Quantitative measurements of GLS in whole, endocardial, and epicardial layers were performed by offline analysis of cine CMR images using commercially available, dedicated feature tracking software (cvi42, Circle, Calgary, Canada, version 5.5). The LV endocardial and epicardial borders were manually delineated in the end-diastolic phase in all short- and long- axes slices where the LV myocardium is intact and visible. The upper septal insertion point of the right ventricle was defined in the short-axis series as an anatomic landmark to allow accurate segmentation of the LV according to a recognised standard model. The extent of the LV myocardium was defined in the long-axis series to define the analysis range. Manual contours were adjusted if there was evidence of poor tracking, whereby the contours deviated from the endocardial and epicardial borders based on visual judgement. GLS was derived from 3 long axis views (2-chamber, 3-chamber, and 4-chamber). cvi42 calculates GLS by tracking the motion of intra-myocardial voxels in the long axis cine images. Endocardial and epicardial GLS values were derived by averaging the peak endocardial and epicardial longitudinal strain values respectively from the 2-chamber, 3-chamber, and 4-chamber slices of the long axis cine images. We used this same methodology for CMR-FT analysis in a previous paper [14]—20 patients from this study cohort of 100, were analysed in this paper focusing on intra- and inter-observer reproducibility of CMR-FT.

### Echocardiography acquisition and 2D speckle tracking analysis

Transthoracic echocardiogram was performed using commercially available machines (GE Vivid 7 and 9). Echocardiographic images were taken at 70–80 frames per second. Routine short axis views at basal, middle, and apical levels of the left ventricle and apical two- and four- chamber and long-axis views were acquired. In STE, only longitudinal strain analysis was performed.

The commercial software Echopac (GE Ultrasound, Haifa, Israel) was used for offline analysis of echocardiographic images. The endocardial and epicardial borders were manually delineated and the speckle tracking algorithm automatically divides the myocardial thickness into three layers of similar thickness, defined as endocardial, mid-myocardial, and epicardial. The software is able to calculate layer-specific global strain values within each layer. A standard 18 segment LV model is utilised by the software. Left ventricular ejection fracture (LVEF) was calculated using the biplane Simpson’s method.

The assessors for speckle tracking and feature tracking were different, with each assessor receiving training on utilising the software and supervision from a cardiac imaging specialist. The assessors were blinded to each other’s results.

Whereas Echopac software permits analysis of myocardial strain in three layers (endocardial, mid-myocardial, and epicardial) as described above, cvi42 software only allows calculation of two layers (endocardial and epicardial). This effectively means that CMR-FT derived endocardial and epicardial strain measurements involve a greater proportion of the myocardium, compared to analogous measurements by 2DSTE. To account for this, we used a weighted average for our STE measurements, such that weighted endocardial strain = [endocardial STE strain + (0.5 × mid-myocardial STE strain) ÷ 1.5]. Similarly, weighted epicardial strain = [epicardial STE strain + (0.5 × mid-myocardial STE strain) ÷ 1.5]. We present our results with both the weighted and non-weighted values.

### Statistical analysis

All statistical analysis was performed using commercially available software, IBM SPSS Statistics 25 (SPSS Inc., Chicago, Illinois, USA). A P < 0.05 was considered statistically significant. Continuous data is expressed as mean ± standard deviation. Agreement was measured using Bland Altman plots. Comparison of agreement was analysed using Pearson’s correlation coefficient, intraclass correlation coefficient, and Fisher r-to-z transformation, with an absolute z-observed value > 1.96 and P < 0.05 considered statistically significant. The paired sample t-test was used to compare the mean difference between the CMR-FT and STE datasets.

## Results: direct comparison of CMR-FT and STE derived whole- and multi-layer global longitudinal strain values

Demographic data of the study cohort is shown in Table 1 below.

**Table 1:** Demographic, clinical, laboratory, and radiographic data of acute myocardial infarction patients (n = 100)

Parameters	AMI patients (n = 100)	
Age	53 ± 17	
Male gender (%)	82	
Body mass index, kg/m^2^	27.5 ± 4.6	
Systolic BP, mmHg	135 ± 27	
Diastolic BP, mmHg	79 ± 16	
Heart rate, bpm	75 ± 20	
Medical History		
Diabetes mellitus	24	
Smoker (current or > 10 pack year history)	58	
Family history of ischaemic heart disease	26	
Hypercholesterolaemia	48	
Hypertension	48	
LV volumes and indices on CMR	**Baseline**	**Follow up**
LV end-diastolic volume, mL	153 ± 34	158 ± 36
LV end-systolic volume, mL	80 ± 26	75 ± 31
LV stroke volume, mL	73 ± 17	54 ± 10
LV mass	134 ± 33	127 ± 26
LV Ejection Fracture (%)	**Baseline**	**Follow up**
STE measurement	49.5 ± 8.5	55.0 ± 10.7
CMR measurement	48.7 ± 8.9	53.8 ± 10.3
Infarction characteristics on CMR		
Baseline myocardial scar core size (5SD)	8.8 ± 6.7	
Follow up myocardial scar core size (5SD)	6.8 ± 5.2	
Negative LV remodeling %	14	
Microvascular obstruction %	30	
Laboratory date		
Admission creatinine, umol/L	85 ± 22	
72 h high sensitivity troponin T ug/L	2.28 ± 1.8	

Continuous data is presented as mean ± standard deviation

The intermodality correlation between whole and multi-layer GLS values derived by CMR-FT and STE are shown in Table 2. The weight endocardial and epicardial strain indicates that a weighted average was applied to the STE derived strain parameters as described in “Echocardiography acquisition and 2D speckle tracking analysis” section  of the Methodology.

**Table 2:** Comparison of global longitudinal strain measurements between CMR-FT and STE using Pearson’s correlation co-efficient, Intraclass correlation co-efficients, and Bland Altman analysis

Strain	CMR-FT	STE	r	ICC	Bias	LOA	P-value
Endocardial	− 15.31 ± 4.1	− 17.10 ± 4.2	0.685	0.626	− 1.79	± 6.4	0.585
Weighted endocardial	− 15.31 ± 4.1	− 16.41 ± 4.0	0.686	0.663	− 1.00	± 6.2	0.780
Whole layer	− 14.33 ± 3.9	− 15.15 ± 3.7	0.687	0.671	− 0.80	± 5.9	0.219
Epicardial	− 14.24 ± 3.6	− 13.26 ± 3.2	0.660	0.632	0.98	± 5.5	0.055
Weighted epicardial	− 14.24 ± 3.6	− 13.85 ± 3.4	0.661	0.657	0.39	± 5.6	0.252

For ICC values, all p < 0.001

Key: bias = mean of differences between CMR-FT and STE measurements, r = Pearson’s correlation co-efficient (all p < 0.001), LOA = limits of agreement; 1.96 × standard deviation of the differences between the two measurements, p-value is that of linear regression on Bland Altman analysis

**Fig. 1:**
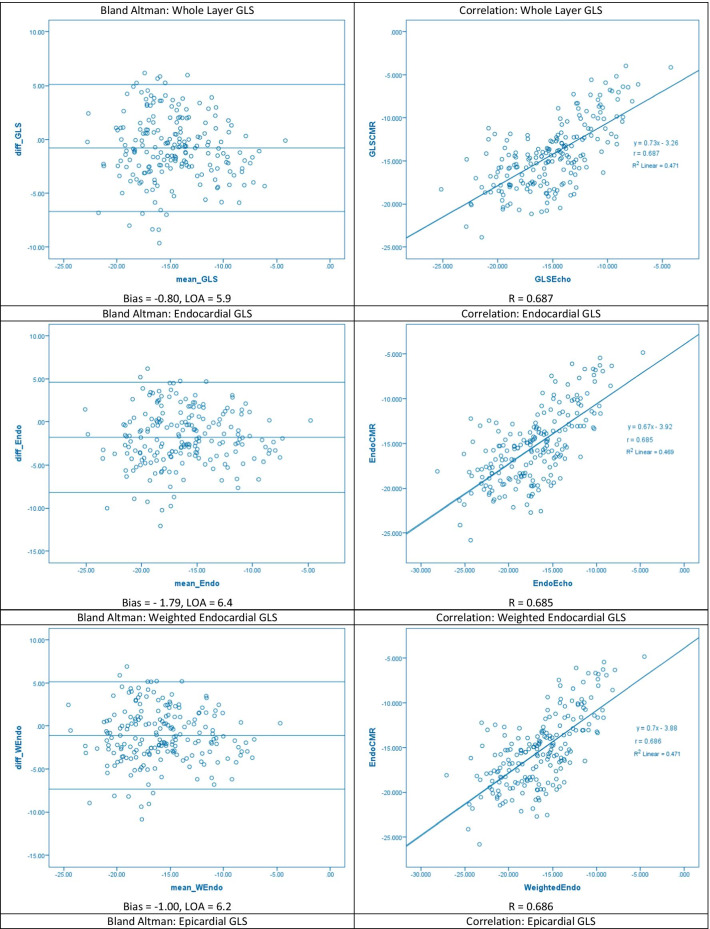

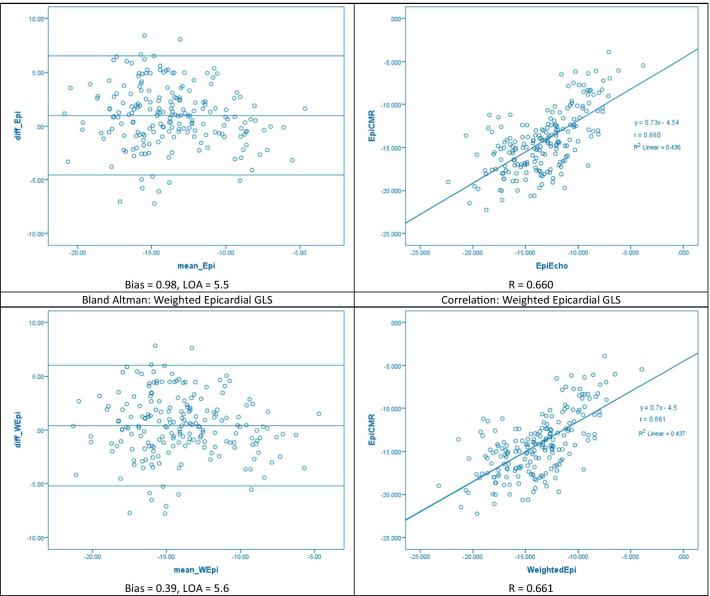
Inter-modality agreement and correlation between CMR-FT and STE derived strain parameters for whole-layer and multi-layer GLS at the endocardial and epicardial borders

The Bland Altman plots and linear correlation graphs are shown in Fig. 1.

### Global longitudinal strain

Overall, good inter-modality correlation was shown for whole-layer GLS with r = 0.687, p < 0.001. The Bland Altman analysis revealed satisfactory agreement with an absolute LOA of 5.9%. Using a paired sample t-test, the mean GLS values assessed by CMR-FT were significantly lower than STE (t = 3.7, p < 0.001).

### Endocardial global longitudinal strain

Endocardial GLS strain values were significantly lower than STE both with (t = 7.8, p < 0.001) and without (t = 4.9, p < 0.001) applying the weight average to the STE-derived measurement. Inter-modality agreement was satisfactory both with (r = 0.686, p < 0.001) and without (r = 0.685, p < 0.001) the weighted average. The Pearson’s correlation co-efficient for endocardial GLS strain values is similar to that of whole-layer GLS. This is explained by the high collinearity between endocardial and whole-layer GLS observed in both imaging modalities (CMR: r = 0.990, p < 0.001, STE: r = 0.997, p < 0.001). Using the weighted endocardial strain measurements showed less bias (-1.00 vs –1.79) and better inter-modality agreement with Bland Altman analysis with smaller LOA [LOA 6.2, p = 0.780 vs LOA 6.4, p = 0.585).

### Epicardial global longitudinal strain

Epicardial GLS strain values derived by CMR-FT were significantly higher than STE both with (t = 4.9, p < 0.001) and without (t = 1.9, p < 0.001) using the weighted average for the STE-derived epicardial strain. Inter-modality correlation in epicardial GLS values was good, and the Pearson’s correlation co-efficient (r = 0.66) was similar to that reported for endocardial (r = 0.685) and whole-layer GLS (r = 0.685) strain values. Bland Altman analysis revealed satisfactory agreement with absolute LOA of 5.6% and 5.5% respectively with and without using the weighted average. Using the weighted average for STE-derived epicardial strain, bias was reduced (0.39 vs 0.98), however limits of agreement were similar.

### Transmural strain gradient

The transmural strain gradient is defined as the difference between the endocardial and epicardial strain values. In CMR-FT, the transmural strain gradient had an absolute mean of 1.07 ± 1.0. In STE derived strain, the gradient was 3.85 ± 1.1 (without weighted average) and 2.56 ± 0.75 (with weighted average). A paired sample t-test showed that the difference in the transmural strain gradient between CMR-FT and STE was statistically significant both without using the weighted average (t = 33.9, p < 0.001) and using the weighted average (t = 21.8, p < 0.001).

## Discussion

The main finding of this study is that there is good intermodality agreement between CMR-FT and STE for the assessment of multilayer global left ventricular longitudinal strain as assessed by Pearson’s correlation coefficient and Bland Altman analysis. Moderate agreement was shown in assessment using the intra-class correlation co-efficient. To the best of our knowledge, this is the first such study in multilayer strain. Previous studies in whole-layer GLS have shown good to excellent intermodality correlation [6, 7] and measurements have been validated against CMR tagging in CMR-FT studies [4, 5, 15] and in CMR tagging and sonomicrometry in STE studies [16].

Similar to previous studies in multilayer strain our results show a clear heterogeneity in the transmural distribution of GLS, with endocardial strain being greater than epicardial strain [12, 13]. The transmural gradient was more pronounced in the STE measurements compared to CMR-FT, both with and without the weighted average. This likely represents differences in software algorithms used to calculate strain, suggesting STE is overestimating endocardial and underestimating epicardial strain relative to CMR-FT. We utilised a weighted average to reconcile the discrepancy between cvi42 and Echopac in measuring multilayer strain, as discussed in the methodology, which decreased the bias and limits of agreement in the Bland Altman plots.

**Fig. 2:**
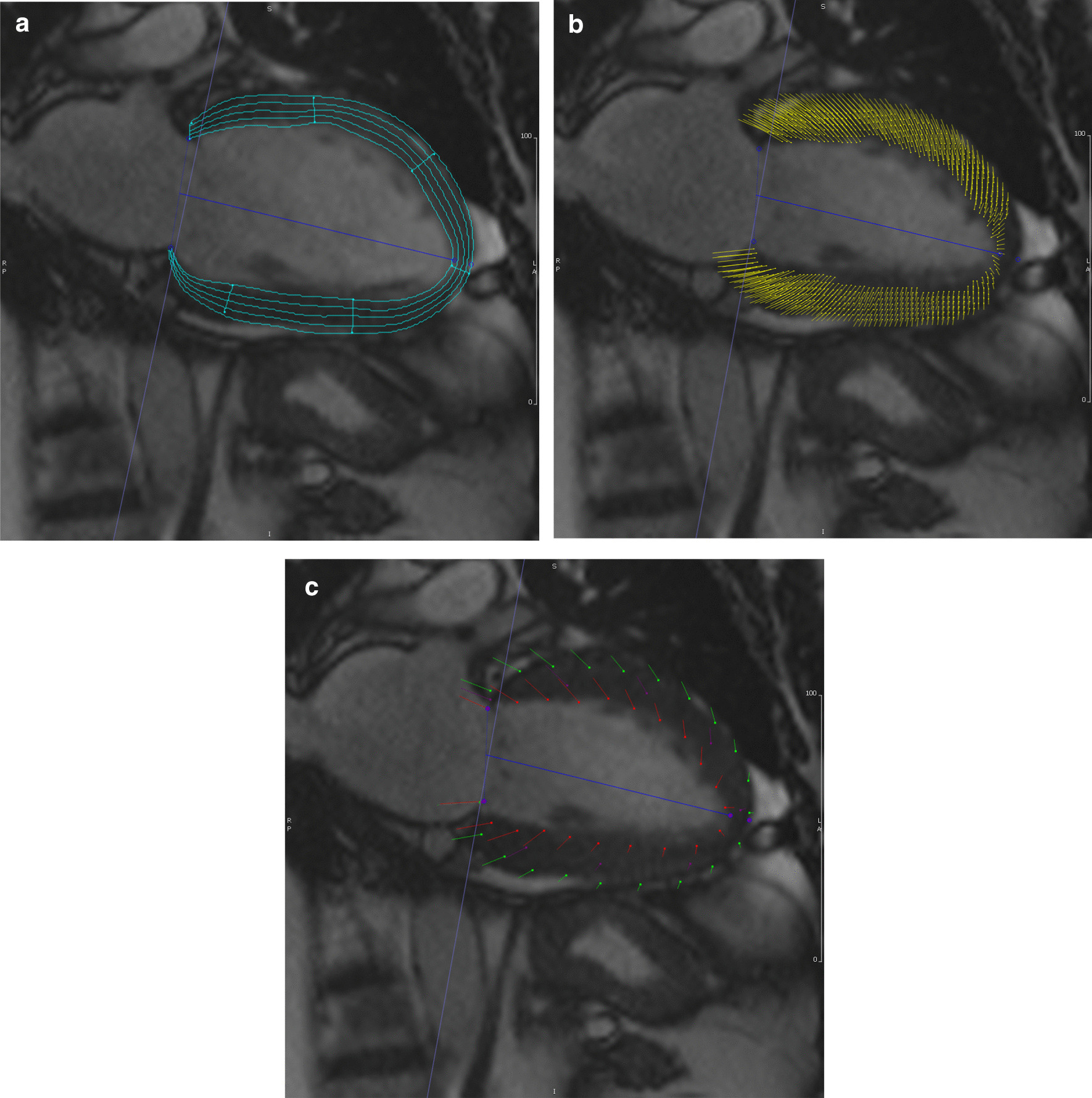
Representative image of CMR feature tracking in long axis 2-chamber views showing **a** multilayer strain, **b** intramyocardial voxels, **c** endocardial and epicardial border delineation

**Fig. 3:**
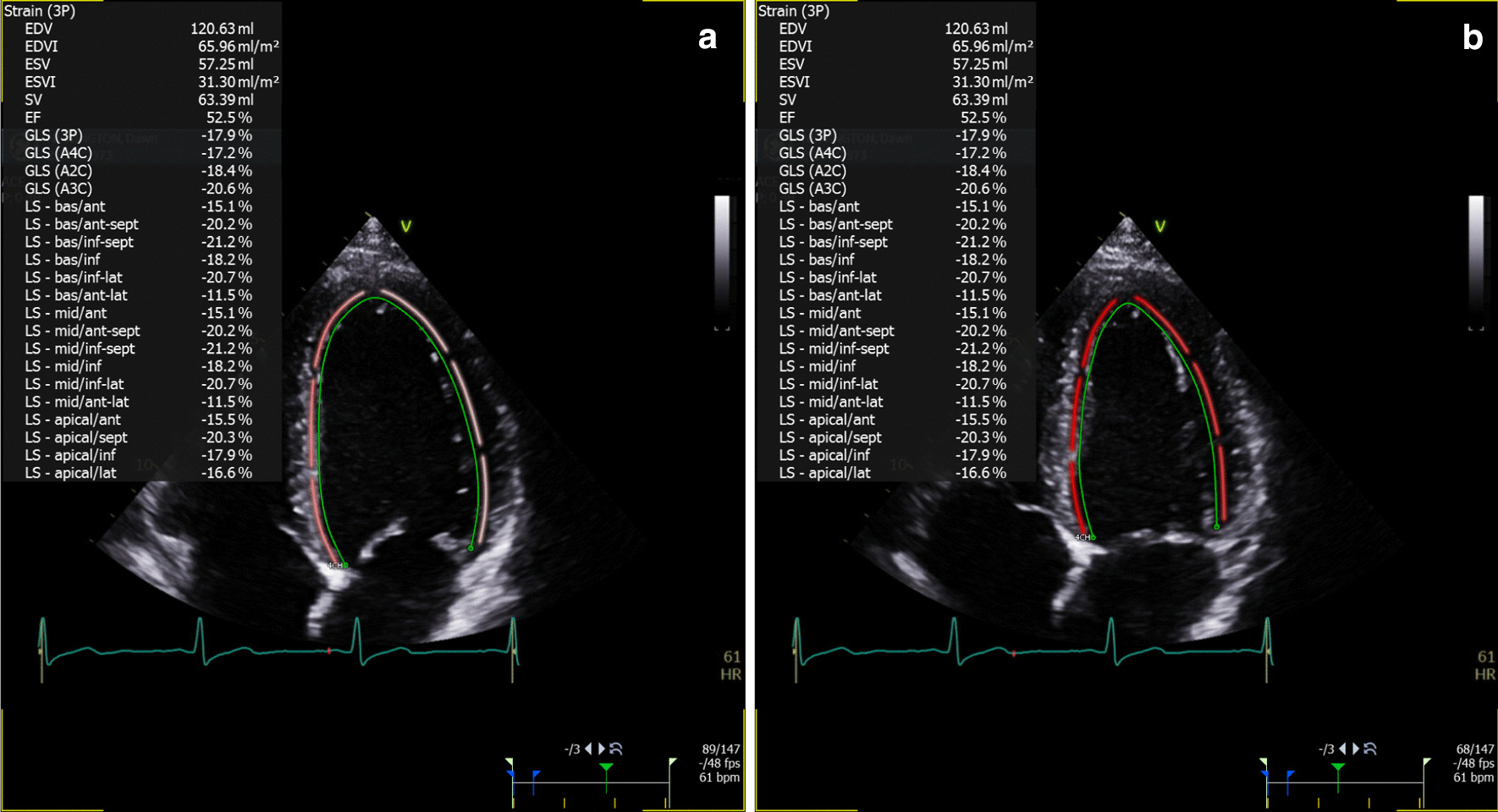
Representative image of STE strain in long axis 2-chamber views

It should be noted that the limits of agreement in our study are wide, but comparable to previous direct comparative studies between CMR-FT and STE derived GLS. Obokata et al. studied 106 patients varied cardiac pathology and reported high correlation coefficient with comparable LOA [6]. Onishi et al. reported high correlation but with higher LOA, in a study of 73 patients with suspected heart failure [7]. Similar to our study, Orwat et al. (n = 40, healthy volunteers and DCM patients) [17] and Kempny et al. (n = 28, Tetralogy of Fallot patients) reported LOA of > 5% for GLS [18]. A recent study by Erley et al. (n = 50) showed good intermodality agreement between whole-layer GLS with comparable correlation coefficients to our study but wider LOA [19]. Pyrds et al. (n = 50) also showed good correlation between CMR-FT and STE-derived whole-layer GLS, but with suboptimal agreement on Bland Altman analysis, limited by a large bias [20]. Each study reported that the absolute value of CMR-FT derived GLS was lower than STE, comparable to our findings, suggesting that the former underestimates GLS compared to the latter. It is worth noting that whilst CMR-FT and STE are similar tissue tracking technologies, they are not identical. Whilst both track the motion of intra-myocardial voxels or speckles, the former also tracks the endocardial and epicardial borders [21]. Pedrizetti proposed that since CMR acquires data over several heart beats, minor beat-to-beat alterations in strain, especially during the rapid isovolumetric phase of contraction, are missed. This effect is dependent upon temporal resolution, and can contribute to underestimation of strain values. An example of tissue tracking in CMR-FT and STE is shown in Figs. 2 and 3 respectively.

The wide limits of agreement and the underestimation of GLS by CMR-FT in these papers suggest that intermodality measurements are not interchangeable. With current discrepancies between the two technologies, serial examination of patients requires a consistent imaging modality to reliably compare readings. Hence, the decision to use STE or CMR-FT should be considered on an individual basis to guide clinical decisions. CMR-FT can be considered in patients with suboptimal echogenic windows in whom speckle tracking analysis is technically difficult. A major limitation of STE analysis is the dependency on image quality [21]—hence in patients with ultrasound dropouts, reverberations, increased field noise, and poor imaging windows, measurements become unreliable. CMR-FT offers advantages over STE in terms of superior image quality as studies report significantly fewer non-analysable segments and larger fields of view. There are additional benefits in terms of superior volumetric analysis. and evaluation of perfusion, microvascular obstruction, and tissue composition. The limitations of CMR over echocardiography are mainly practical, as the latter is more cost-effective, accessible, repeatable, and can be used as a bedside diagnostic tool.

### Clinical implications

We have previously reported that multilayer GLS and GCS measurements demonstrate excellent intra-observer and inter-observer reproducibility in CMR-FT [14]. The significance of excellent inter-reproducibility is that CMR-FT can be incorporate in clinical practice where clinicians of varying expertise may be analysing patient scans. Subtle changes in endocardial and epicardial strain can be reliably determined using serial examinations. This study combined with our previous findings show that CMR-FT can be reliably utilised in settings where appropriate as a strain imaging modality.

Layer-specific approaches to strain imaging can provide additive diagnostic and prognostic value when compared to whole-layer strain analysis and conventional echocardiographic indices such as LVEF. Longitudinal strain is more sensitive to the initial stages of cardiac disease, which often affect subendocardial myocardial fibres to a greater extent. Endocardial GLS has potential for use as a risk stratification tool to aid early diagnosis of cardiomyopathies and coronary artery disease, allowing for initiation of patient-centric therapeutic interventions and reduction of morbidity. Ozawa et al. reported that endocardial GLS was disproportionately affected in HOCM, whereas endocardial GCS was maintained [11]. GCS is more likely to be impaired in patients with longstanding cardiac pathology due to the transmural spread of the disease towards the mid-myocardium and subepicardium. Endocardial GLS was also shown to be disproportionately affected in NSTEMI patients compared to epicardial strain and LVEF, and is a superior index in identifying significant coronary artery disease [10]. A recent study by Tanacli et al. established CMR-FT derived epicardial GLS along with NT-proBNP as a viable composite predictive tool in the diagnosis of HFpEF with excellent specificity and sensitivity [22]. NT-proBNP alone has limitations in detecting early HFpEF and cannot reliably discriminate between HFpEF and HFrEF. However, strain parameters are differentially affected in these disease states, with endocardial GCS being preserved in HFpEF and significantly reduced in HFrEF [23]. Hence, endocardial GLS and GCS can be incorporated to reliably discriminate between healthy individuals, early HFpEF, and HFrEF. The prognostic value of GLS has been reported in the literature, but is limited for multilayer strain. Earlier studies have shown superiority in predicting major adverse cardiac events compared to LVEF and CMR markers of infarct severity [3, 24] in both STEMI and other cardiac disease states. Currently there is no consensus on defining cut-off values that correlate with hard endpoints, which are needed to facilitate informed clinical decision making. This will continue to be an area of interest within the field of strain imaging, and represents a much-needed focus for future research.

### Limitations

This study has some limitations. Firstly, due to software differences our cvi42 measurements were limited to two myocardial layers, compared to the Echopac measurements which include the mid-myocardial layer. The study introduced a weighted average to reconcile this discrepancy, though this is not feasible for clinical practice. Future directions in strain imaging should look toward introducing greater uniformity in multilayer strain measurement.

Secondly, strain analysis was performed using only a single software. The literature shows that inter-vendor reproducibility is a limiting factor for routine implementation of CMR-FT in clinical practice, with a recent study by Heinke et al. highlighting the need for standardised postprocessing of GLS measurements in reducing variability [25]. Although this study focusses on global strain, the issue of intervendor variability has also been reported in measuring multi-layer segmental strain [26].

Thirdly, our study focusses solely on global longitudinal strain. Previous studies have shown that multilayer GCS also has important clinical applications and can provide additive diagnostic value in assessing infarct transmurality [27] and estimating functional recovery in ischaemic heart disease [28]. The endocardial-to-epicardial GCS strain gradient was also shown to discriminate between different transmurality categories in AMI [29]. In a study focusing on multilayer STE-derived GCS, Zhang et al. showed that reductions in subendocardial strain can represent early ischaemic changes, with potential for use as a risk stratification tool [30]. To the best of our knowledge the inter-modality agreement of multilayer GCS between CMRT-FT and STE has not been investigated, and represents an area for future discussion.

Furthermore, it is important to recognise that our study consists entirely of AMI patients, and future direct comparison studies in multilayer strain need to incorporate healthy volunteers to provide generalisability of data to a wider population. Finally, it is important to recognise that CMR-FT multilayer strain data has not been validated against gold standard modalities such as sonomicrometry or CMR tagging, which represents another missing piece within the literature.

### Conclusion

Although not interchangeable, CMR-FT demonstrates good inter-modality agreement with STE for the measurement of layer specific global longitudinal strain values using Bland Altman analysis.

## Data Availability

The datasets generated and/or analysed during the current study are not publicly available as this requires additional permission from the department but are available from the corresponding author on reasonable request.

## Abbreviations

AMI: Acute myocardial infection
CMR: Cardiac magnetic resonance
CMR-FT: Cardiac magnetic resonance feature tracking
GCS: Global circumferential strain
GLS: Global longitudinal strain
LV: Left ventricle
HFpEF: Heart failure with preserved ejection fracture
HOCM: Hypertrophic obstructive cardiomyopathy
ICC: Intraclass correlation co-efficients
LVEF: Left ventricle ejection fraction
NSTEMI: Non-ST elevation myocardial infarction
STE: Speckle trackle echocardiography
STEMI: ST elevation myocardial infection
